# Nationally recruited telehealth group-based Cognitive Behavioral Therapy for Insomnia in patients with primary brain tumors: A randomized NIH ORBIT Phase IIc study

**DOI:** 10.1093/noajnl/vdag226

**Published:** 2026-08-31

**Authors:** Ashlee R Loughan, Autumn Lanoye, Kelcie D Willis, Amber Fox, Mary Bridgman, Nolan Wages, Youngdeok Kim, Alicia Zukas, Christopher Kleva

**Affiliations:** School of Medicine, Virginia Commonwealth University, Richmond, Virginia, USA; Massey Comprehensive Cancer Center, Virginia Commonwealth University, Richmond, Virginia, USA; School of Public Health, Virginia Commonwealth University, Richmond, Virginia, USA; Department of Psychiatry, Massachusetts General Hospital, Boston, Massachusetts, USA (K.D.W.); Department of Psychology, Virginia Commonwealth University, Richmond, Virginia, USA; School of Medicine, Virginia Commonwealth University, Richmond, Virginia, USA; Massey Comprehensive Cancer Center, Virginia Commonwealth University, Richmond, Virginia, USA; Massey Comprehensive Cancer Center, Virginia Commonwealth University, Richmond, Virginia, USA; School of Public Health, Virginia Commonwealth University, Richmond, Virginia, USA; Department of Kinesiology and Health Sciences, Virginia Commonwealth University, Richmond, Virginia, USA; Department of Neurosurgery, Medical University of South Carolina, Charleston, South Carolina, USA (A.Z.); Department of Psychology, Virginia Commonwealth University, Richmond, Virginia, USA

**Keywords:** cognitive behavioral therapy for insomnia, insomnia, primary brain tumor, randomized trial, sleep disturbance

## Abstract

**Background:**

Sleep disturbance is common and undertreated in adults with primary brain tumors. Cognitive Behavioral Therapy for Insomnia (CBT-I) is the first-line, non-pharmacological treatment, yet its benefit in neuro-oncology has yet to be evaluated. This Phase IIc pilot assessed feasibility, acceptability, and meaningful effects of telehealth group-based CBT-I versus treatment as usual (TAU) in neuro-oncology.

**Methods:**

Adults with primary brain tumors and chronic insomnia were randomized 1:1 to 6 weekly CBT-I sessions or TAU. Primary outcomes were feasibility of recruitment and longitudinal data collection, acceptability of CBT-I and study procedures, and changes in patient-reported insomnia severity, fatigue, and sleep quality.

**Results:**

Recruitment met a priori feasibility benchmarks: Of the 84 patients screened, 96% were eligible, 86% consented, and 60 were randomized (CBT-I *n *= 29; TAU *n *= 31). Baseline data collection exceeded benchmarks, while longitudinal data collection was variable across outcomes. Patients deemed CBT-I as acceptable (86% intervention retention, 100% reporting improved sleep, 100% patient recommendation) and provided high satisfaction ratings (>80%) across delivery, compensation, and most study procedures. On descriptive comparisons, CBT-I demonstrated greater preliminary acute improvements in insomnia severity (61% vs. 33%), fatigue (57% vs. 35%), and sleep quality (74% vs. 30%) than TAU, with medium-to-large effects (Cohen’s *d*= −0.69 to −1.63 vs. *d*= −0.33 to −0.73) observed on continuous measures.

**Conclusions:**

Telehealth CBT-I is feasible, acceptable, and shows a promising benefit signal in adults with primary brain tumors, particularly for improving insomnia. Future trials should optimize procedures for neurocognitive and accelerometer longitudinal data collection to support fully powered efficacy studies.

**Trial Registration:**

ClinicalTrials.gov Identifier: NCT06439420.

Key Points

- CBT-I is a treatment for sleep, with limited evidence in patients with PBT.
- Results of this study suggest CBT-I is feasible and acceptable in patients with PBT and comorbid insomnia.
- Benefits in subjective insomnia, sleep quality, and fatigue were found.

Importance of the Study

Sleep disturbance is common yet often underassessed and undertreated in adults with primary brain tumors (PBT). Although Cognitive Behavioral Therapy for Insomnia (CBT-I) is the first-line treatment for chronic insomnia and has demonstrated efficacy across medical and oncology populations, individuals with PBT have largely been excluded from prior trials. This Phase IIc is the first randomized pilot trial to evaluate telehealth-delivered, group-based CBT-I vs. treatment as usual (TAU) in adults with PBT and chronic insomnia. Findings demonstrate that delivering CBT-I in this population is feasible and acceptable, supporting the practicality of conducting behavioral sleep interventions in neuro-oncology. Descriptive comparisons also suggest greater improvements in insomnia severity, sleep quality, and fatigue among patients receiving CBT-I compared with TAU. These findings highlight the potential of CBT-I to address a significant symptom burden and provide a strong rationale for fully powered randomized controlled trials in this vulnerable population.

Sleep disturbance is among the most prevalent and burdensome symptoms reported by those diagnosed with PBT, defined here as tumors of the brain parenchyma, sella, and meninges.^1-5^ Across studies, up to 60% of patients with PBT report comorbid symptoms of insomnia,^6^ with approximately 25% meeting criteria for clinical severity,^7^^,^^8^ a rate substantially higher than that observed in the general population.^9^ Patients with PBT also report excessive daytime sleepiness and fatigue^10-12^ and, altogether, these symptoms are associated with impairments in daily functioning, reduced vocational participation, increased healthcare utilization, diminished patient independence, and reduced life quality.^6^^,^^13-15^ In this population, disrupted sleep may also exacerbate seizure risk, with downstream consequences for cognitive function, mood, functional status, and survival.^16-18^ Despite the clinical impact of these symptoms, insomnia in those with PBT frequently goes unassessed and inadequately treated,^19^ highlighting a critical gap in supportive neuro-oncology care.

Pharmacologic treatments for insomnia are often limited in patients with PBT due to concerns regarding cognitive side effects, psychomotor slowing, tolerance, and unsuitability for long-term use.^19-22^ Cognitive Behavioral Therapy for Insomnia (CBT-I), the first-line treatment for chronic insomnia,^23^ offers a non-pharmacological alternative with robust empirical support across medical and psychiatric populations, including those with cancer.^24^^,^^25^ CBT-I combines behavioral, cognitive, and educational strategies to reduce conditioned arousal and maladaptive sleep-related behaviors^26^ and has demonstrated efficacy when delivered in group-based^27^ and telehealth formats.^28^ Although CBT-I is recommended in the National Comprehensive Cancer network (NCCN) guidelines for cancer-related fatigue,^29^ patients with PBT have been largely excluded from behavioral intervention trials, which may partly reflect concerns regarding neurocognitive impairment in this population. However, our preliminary data indicate that most patients (91%) pass commonly utilized cognitive screeners^30^ and are capable of meaningful participation. Further, CBT-I has shown benefit in other populations with mild cognitive deficits.^31^ Lastly, in our team’s recent single-arm proof-of-concept trial, patients with PBT described the intervention as beneficial and endorsed improvements in subjective insomnia severity, sleep quality, and fatigue with large effect sizes;^32^ however, the intervention’s feasibility, acceptability, and potential for clinical benefit have yet to be evaluated in a larger, randomized-controlled trial.

This Phase IIc trial was designed in accordance with the NIH ORBIT model for developing behavioral interventions.^33^ The NIH ORBIT model aligns closely with the process of drug development by taking a progressive, four-phase approach to demonstrate that behavioral treatments can improve chronic diseases or mental health conditions with scientific rigor. These phases are: (1) *Design*, which aims to assemble and refine the basic features of a behavioral treatment (eg identifying risk factors or clinical endpoints, developing or refining intervention components, etc.); (2) *Preliminary Testing*, which aims to demonstrate whether a trial can be successfully conducted and provide initial plausibility that a specific treatment can improve identified clinical endpoints (eg adequate recruitment or enrollment rates, trial process criteria, and reduction of symptom severity). Furthermore, these identified endpoints are iteratively examined across proof-of-concept trials, feasibility trials, and small efficacy pilot trials; (3) *Efficacy*, which aims to definitively determine that a behavioral treatment can produce a clinically or statistically significant effect on an important disease outcome when compared to a control condition and/or another treatment; and, (4) *Effectiveness*, which aims to determine whether the behavioral treatment can be implemented into real-world clinical practice outside of the confines of a controlled research trial.

Accordingly, building on our proof-of-concept single-arm trial of CBT-I,^32^ this study is the first randomized controlled evaluation of CBT-I versus treatment as usual (TAU) in adults with PBT and comorbid insomnia. The primary aims of this Phase IIc trial were to evaluate: (1) the feasibility of recruitment, enrollment, and longitudinal data collection, (2) the acceptability of CBT-I and study procedures, and (3) meaningful benefits on patient-reported outcome measures (PROMs) of insomnia severity, fatigue, and sleep quality. Informed by a priori feasibility and acceptability benchmarks,^32^^,^^34-38^ we hypothesized that: (1a) recruitment and enrollment would be feasible, (1b) data collection would be feasible across study procedures and timepoints, (2a) CBT-I would be acceptable, (2b) study procedures would be acceptable to participants across arms, and (3) a greater proportion of CBT-I participants would report meaningful benefit from baseline to post-treatment (Week 6) with respect to insomnia severity, fatigue, and sleep quality compared to TAU participants.

## Methods

### Procedures

The current study was approved by the ethics committee of Virginia Commonwealth University (VCU) and Massey Comprehensive Cancer Center (HM20028336) and registered with ClinicalTrials.gov (NCT06439420). Patients with a diagnosis of PBT were recruited using a multimodal approach: local recruitment (eg physician referral, direct mailer/email) and nationwide recruitment via patient-facing speaking engagements (eg conferences; webinars), social media (eg digital fliers; brief animated recruitment video), and engaging other providers at cancer centers across the United States. Interested individuals contacted the VCU LiveNOW (Neuro-Oncology Wellness) Lab to express interest in the trial and were subsequently screened for eligibility by a trained research coordinator. After 3 failed attempts to contact interested individuals, they were marked as lost to follow-up (LTFU). Informed consent was obtained for those who met eligibility criteria and were willing to participate in the study.

After obtaining consent, participants were stratified by corticosteroid dose (ie dexamethasone >6 mg/day or prednisone >40 mg/day v. dexamethasone <6 mg/day or prednisone <40 mg/day), where applicable, and then randomized in a 1:1 ratio to CBT-I or TAU. Randomization employed a block scheme with a block size of 4 to ensure balanced arm assignments within each stratum after every four participants. The randomization sequence was generated and uploaded to REDCap for secure allocation (Figure 1). Participants assigned to CBT-I completed six weekly 90-minute group CBT-I sessions delivered via telehealth by trained study interventionists (advanced graduate trainees in clinical psychology: AMF and CSK). Telehealth groups did not exceed 5 participants. CBT-I sessions were conducted using a secure videoconferencing platform. Participants randomized to TAU received a sleep hygiene handout and links to online mental health resources but did not receive group telehealth CBT-I from the LiveNOW study team. All participants completed assessments at baseline (Week 0; pre-randomization), post-intervention (Week 6), and follow-up (Week 18). Self-reported demographic and medical information were collected at baseline using a secure online data capture system (REDCap).^39^ PROMs were administered electronically, using the same secure online data capture system, at all 3 timepoints. Objective sleep data were collected using a wrist-worn accelerometer (ActiGraph GT9X), worn for seven consecutive days at each assessment period; concurrently, participants also completed a daily subjective sleep diary (Consensus Sleep Diary-Core).^40^ Accelerometers and sleep diaries were shipped using the United States Postal Service (USPS) with return postage included. A brief 30-min neurocognitive battery was also administered at Weeks 0, 6, and 18. For the CBT-I group, participants additionally completed a brief survey assessing intervention satisfaction at post-intervention (Week 6), and all participants completed a brief satisfaction survey assessing longitudinal data procedures at follow-up (Week 18). Participants were contacted via email up to 3 times for all data collection procedures (PROMs, accelerometry, neurocognitive testing), across all 3 timepoints. Post-intervention and follow-up assessments (Weeks 6 and 18) not completed within 1 month were considered LTFU.

**Figure 1.**
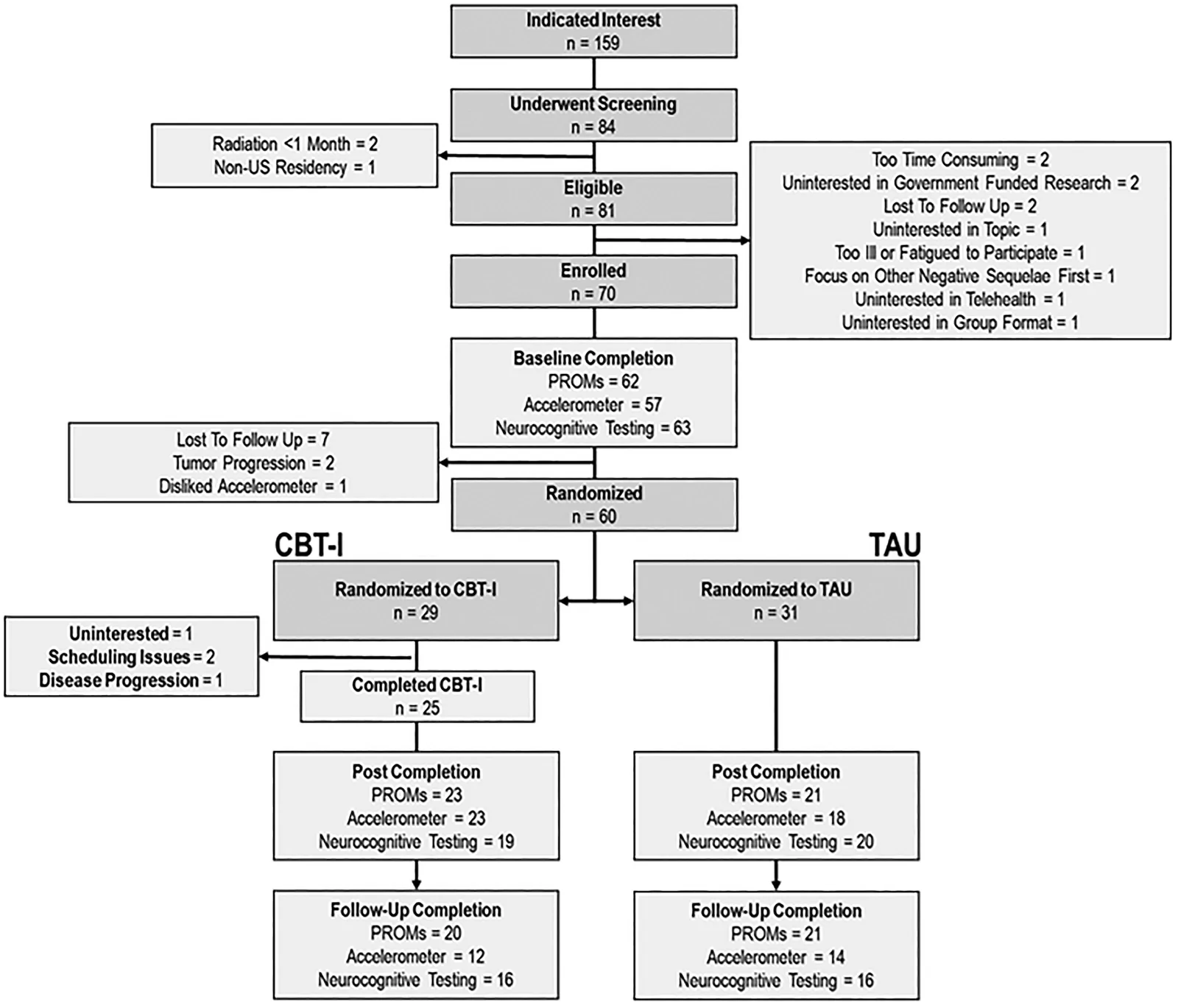
Study CONSORT diagram.

Study interventionists were trained to deliver CBT-I using a structured, three-stage training model. *Stage 1* focused on foundational education in neuro-oncology, including didactic instruction on disease characteristics, treatment modalities, common side effects, and patient and family stressors. All interventionists had >24 months of prior exposure to neuro-oncology populations. *Stage 2* consisted of independent review of the CBT-I treatment manual and completion of a one-day intensive CBT-I training workshop facilitated by CBT-I consultant (KDW). *Stage 3* required interventionists to complete at least one full CBT-I case with a mock patient prior to certification of competency. All mock sessions were video recorded and reviewed, with individualized feedback provided by KDW. Ongoing fidelity and clinical support were maintained through weekly supervision sessions. These meetings focused on participant progress, clinical questions, session preparation, and treatment refinement and were led by KDW and/or ARL.

### CBT-I Intervention

CBT-I is an evidence-based structured intervention with multiple treatment components, including stimulus control, sleep restriction training, psychoeducation, sleep hygiene, relaxation training, and cognitive restructuring.^41^^,^^42^ Stimulus control was introduced as a way to reduce conditioned arousal by re-associating the bed with sleep and eliminating associations with other behaviors (eg eating, watching television).^43^ For example, participants are encouraged to get out of bed if they are unable to sleep after 15-20 minutes, returning only when sleepy again. Sleep restriction, also known as sleep efficiency training, is a method for consolidating fragmented sleep by limiting participants’ time in bed to their average sleep time (no less than 5 hours).^43^ After establishing a fixed bedtime and waketime, participants’ bedtime is manipulated each week based on adherence, average sleep efficiency (total sleep time divided by total time in bed), and their subjective need for sleep. As participants’ sleep efficiency approaches 85%, their time in bed is increased in 15-to-30-minute increments until sleep duration, efficiency, and quality are optimized. Psychoeducation includes topics such as a typical night of sleep, causes of insomnia, sleep drive, and circadian rhythm. Examples of sleep hygiene recommendations include avoiding daytime naps, limiting caffeine later in the day, and refraining from worrying in bed.^43^ Foundational principles of CBT, including relaxation training and cognitive restructuring, are used to reduce the impact of stress on sleep.^43^ Intervention adaptations for adults with PBT included tailoring psychoeducation (PBT treatment as a precipitating event, prevalence of insomnia in PBT) and other CBT-I components in the context of the effects of a PBT and its treatment (seizure risks, sleep restriction, stimulus control). See Table 1 for a session-by-session breakdown of the CBT-I intervention.

**Table 1.** CBT-I intervention components^43^

Session	Objectives
1	▪ Introduction; orient participants to the group ▪ Provide psychoeducation on sleep, causes of insomnia, and brain tumor effects ▪ Review sleep diary
2	▪ Introduce stimulus control; problem solve anticipated obstacles ▪ Introduce sleep restriction therapy; set bedtime and waketime ▪ Introduce sleep hygiene guidelines; problem solve anticipated obstacles
3	▪ Review sleep diary progress and adherence; set new bedtimes as appropriate ▪ Provide psychoeducation on stress and its relation to sleep ▪ Introduce relaxation strategies (eg deep breathing, progressive muscle relaxation)
4	▪ Review sleep diary progress and adherence; set new bedtimes as appropriate ▪ Provide psychoeducation on unhelpful thoughts and worry ▪ Introduce cognitive restructuring for managing cognitive distortions
5	▪ Review sleep diary progress and adherence; set new bedtimes as appropriate ▪ Provide psychoeducation on unhelpful sleep-related thoughts ▪ Identify and challenge common myths about insomnia
6	▪ Review sleep diary progress and adherence; set new bedtimes as appropriate ▪ Summarize material covered and skills learned ▪ Create a relapse prevention plan

### Participants

#### Inclusion Criteria

(1) self-report diagnosis of PBT (grade I-IV); (2) age ≥18 years; (3) ability to participate in group sessions via telehealth; (4) met diagnostic criteria for insomnia using the 4-item Structured Clinical Interview for DSM-V Sleep Disorders (SCID) insomnia module.^44^

#### Exclusion Criteria

(1) severe cognitive impairment at the level of a dementia as measured by <21 on the Telephone Interview for Cognitive Status (TICS)^45^—this procedure allowed for the inclusion of patients with mild neurocognitive deficits, which is common in those with brain tumors; (2) cranial surgery within the previous 2 weeks; (3) completion of radiation therapy within the past month, to allow for recovery from acute treatment-related fatigue; (4) major communication difficulties that would prohibit effective intervention interactions as noted by the medical or research study team; (5) were unable to provide informed consent. Patients undergoing chemotherapy or tumor-treatment fields were not excluded. Further, in the event of new onset or ongoing seizure activity and/or disease progression during the intervention, continued patient participation versus withdrawal was determined collaboratively by the study PI (ARL), CBT-I interventionists (AMF, CSK), patient, and medical consultant (AZ).

### Measures

A description of the primary study outcome measures (feasibility and acceptability) is provided in Table 2. In addition, the current study explored meaningful changes in patient-reported sleep outcomes on insomnia severity, fatigue, and sleep quality. Additional exploratory outcomes, including objective sleep measured via accelerometer, patient-reported mood and life quality, and neurocognitive function measured via objective neuropsychological tests will be described and disseminated in a subsequent report.

**Table 2.** Primary study outcomes, metrics, and benchmarks

Outcome		Metric and Benchmark	Metric Timepoints		
			Baseline [0 weeks]	Post [6 weeks]	Follow-Up [18 weeks]
Feasibility	Screening Rates	Proportion of interested patients who were screened (≥70%)	X		
	Eligibility Rates	Proportion of screened patients who were deemed eligible (≥70%)	X		
	Enrollment Rates	Proportion of eligible patients who enrolled in the study (≥40%)	X		
	Completion of PROMs*	Baseline data completion of those enrolled (≥80%) Post-intervention data completion of those randomized (≥70%) Follow-up data completion of those randomized (≥60%)	X	X	X
	Agreement to Accelerometer Participation**	Baseline willingness of those enrolled (≥80%) Post-intervention returned of those randomized (≥70%) Follow-up returned of those randomized (≥60%)	X	X	X
	Successful Receipt & Return of Accelerometer	Baseline receipt & returned of those enrolled (≥80%) Post-intervention receipt & returned of those randomized (≥70%) Follow-up receipt & returned of those randomized (≥60%)	X	X	X
	Adherence of Accelerometer	≥3 days worn at baseline of those enrolled & returned (≥80%) ≥3 days worn at post of those randomized & returned (≥70%) ≥3 days worn at follow-up of those randomized & returned (≥60%)	X	X	X
	Completion of Neurocognitive Testing*	Baseline testing completion of those enrolled (≥80%) Post-intervention testing completion of those randomized (≥70%) Follow-up testing completion of those randomized (≥60%)	X	X	X
Acceptability	Intervention Retention***	Proportion of participants who completed the 6-week intervention (≥80%), inclusive of make-ups		X	
	Session Attendance***	Proportion of attended CBT-I sessions requiring make-ups (<20%)		X	
	Satisfaction Ratings***	Perceived improvement in sleep (≥7; range 1-10) Perceived adherence to CBT-I skills (≥7; range 1-10) Satisfaction with the telehealth format (≥4; range 1-5)		X X X	
	Program Recommendation***	≥75% of patients recommend the program to others (yes/no)		X	
	Study Procedures	Ease of PROM data completion, time commitment, and effort (≥3; range 1-5)			X
		Ease of accelerometer return and adherence, time commitment, and effort (≥3; range 1-5)			X
		Ease of neurocognitive testing completion, time commitment, and effort (≥3; range 1-5)			X
Sleep Related Outcomes	Insomnia Severity	≥6 point score reduction^47^ on the ISI^46^	X	X	X
	Fatigue	≥1.33 point score reduction^49^ on the BFI^48^	X	X	X
	Sleep Quality	≥3 point score reduction^51^ ^,^ ^52^ on the PSQI^50^	X	X	X

PROM = Patient-Reported Outcome Measure, *measured as any completion of data at specified timepoint, **agreement was required due to cost of device prior to shipment, participants who did not respond after three emails were considered lost to follow-up and were not sent an accelerometer, ***assessed only in those randomly assigned to the Cognitive Behavioral Therapy for Insomnia (CBT-I) intervention group; ISI: Insomnia Severity Index; BFI: Brief Fatigue Inventory; PSQI: Pittsburgh Sleep Quality Index.

#### Insomnia Severity

The Insomnia Symptom Inventory (ISI) was used to measure the current frequency and intensity of participants’ insomnia symptoms.^46^ The ISI consists of 7 items rated on a 5-point numerical rating scale. Items are summed to produce a total score ranging from 0 to 28 with higher scores representing greater insomnia severity. The cutoff scores for the ISI are as follows: 0-7 (no clinically significant insomnia symptoms), 8-14 (sub-threshold insomnia), 15-21 (clinical insomnia-moderate severity), and 22-28 (clinical insomnia-severe). A 6-point change in total score is considered to be clinically meaningful.^47^ Cronbach’s alpha on the ISI among previous samples of patients with PBT was found to be .90.^19^

#### Fatigue

The Brief Fatigue Inventory (BFI) was used to measure the severity and impact of fatigue within the past 24 hours.^48^ The BFI consists of 9 items rated on a numeric rating scale from 0 to 10, with higher scores indicating higher levels of fatigue. The total score on the BFI is calculated using the mean from all items. The suggested cutoff scores for the BFI are as follows: 1-3 (mild), 4-7 (moderate), and 8-10 (severe). A 1.33-point change in total score was recently shown to be clinically meaningful in a neuro-oncology population.^49^ Developed for use in oncology, the BFI demonstrates a high internal consistency, with a Cronbach’s alpha of .96 among cancer patients.^48^

#### Sleep Quality

The Pittsburgh Sleep Quality Index (PSQI) was used to measure sleep quality over the past month.^50^ The PSQI consists of 19 items and produces 7 component scores: (1) sleep duration, (2) sleep disturbance, (3) sleep onset latency, (4) daytime dysfunction due to sleepiness, (5) sleep efficiency, (6) overall sleep quality, and (7) sleep medication use. Component scores range from 0 to 3 with higher scores representing greater sleep dysfunction. Component scores are summed to produce a global score ranging from 0 to 21. A global score above 5 has been identified as the cutoff point separating good sleepers from poor sleepers, and a 3-point change in total score is considered to be clinically meaningful.^51^^,^^52^ Cronbach’s alpha on the PSQI in another sample of patients with PBT was .72.^19^

### Data Analytic Plan

To evaluate the feasibility of recruitment and longitudinal data collection as well as the acceptability of CBT-I and study procedures, we used descriptive statistics. Consistent with NIH ORBIT Phase IIc pilot trials, a sample of approximately 60 participants allowed for estimation of feasibility parameters with reasonable precision.^53^ To evaluate acute changes in sleep-related PROMs (insomnia severity, fatigue, and sleep quality) from baseline to post, change (Δ) in total scores were used. To assess for individual-level clinically meaningful change, we used published thresholds to detect minimally important difference (MID; Table 2). Standardized effect sizes were calculated to characterize the magnitude of within-group change from Week 0 to Week 6. Cohen’s *d* (0.2 = small, 0.5 = medium, and 0.8 = large effect) was computed using the observed mean paired difference between Week 0 and Week 6 divided by the standard deviation of the paired differences (ie standardized mean change for paired data). To explore sustained change (Week 6 to Week 18) in those who saw initial benefit from the intervention, change (Δ) in total scores were descriptively examined.

### Results

#### Feasibility of Recruitment

Recruitment occurred from July 2024 to April 2025; 159 individuals indicated interest, 84 participated in screening (53% screening rate), and 81 met study eligibility (96% eligibility rate; Figure 1). Of the 70 eligible individuals who consented to participate (86% enrollment rate), 15 (21.4%) were recruited through local methods and 55 (78.6%) were recruited via national methods. In total, 60 participants were successfully randomized (1:1) to either CBT-I (*n *= 29) or TAU (*n *= 31; Figure 1). See Table 3 for demographic, medical, and psychological variables of those who were randomized. Baseline characteristics were similar between groups.

**Table 3.** Demographic and clinical characteristics of randomized participants at baseline (*N *= 60)

	Overall Sample *n *= 60	CBT-I Sample *n *= 29	TAU Sample *n *= 31
Age (Mean, SD)	51.4 (13.64)	52.9 (15.38)	50.0 (11.87)
Gender (*n*, %)			
Female	31 (51.7%)	14 (48.3%)	17 (54.8%)
Male	26 (43.3%)	14 (48.3%)	12 (38.7%)
Non-Binary	1 (1.7%)	1 (3.4%)	0 (0.0%)
Declined to Disclose	2 (3.3%)	0 (0.0%)	2 (6.5%)
Race/Ethnicity (*n*, %)			
African American/Black	0 (0.0%)	0 (0.0%)	0 (0.0%)
American Indian/Alaskan Native	1 (1.7%)	0 (0.0%)	1 (3.2%)
Asian	3 (5.0%)	3 (10.3%)	0 (0.0%)
Native Hawaiian/Other Pacific Islander	0 (0.0%)	0 (0.0%)	0 (0.0%)
White, Hispanic/Latino	2 (3.3%)	1 (3.4%)	1 (3.2%)
White, Not Hispanic/Latino	48 (80.0%)	23 (79.3%)	25 (80.6%)
Multiple Races	3 (5.0%)	1 (3.4%)	2 (6.5%)
Black/White	1 (1.7%)	0 (0.0%)	1 (3.2%)
Asian/White/Other	1 (1.7%)	0 (0.0%)	1 (3.2%)
White/Declined to Disclose	1 (1.7%)	1 (3.4%)	0 (0.0%)
Other	0 (0.0%)	0 (0.0%)	0 (0.0%)
Declined to Disclose	2 (3.3%)	0 (0.0%)	2 (6.5%)
Missing	1 (1.7%)	1 (3.4%)	0 (0.0%)
Highest Level of Educational Attainment (*n*, %)			
Less than High School	1 (1.7%)	0 (0.0%)	1 (3.2%)
High School Degree/Equivalent	2 (3.3%)	1 (3.4%)	1 (3.2%)
College	57 (95.0%)	28 (96.6%)	29 (93.5%)
Some Courses	12 (20.0%)	9 (31.0%)	3 (9.7%)
2-year Degree	7 (11.7%)	5 (17.2%)	2 (6.5%)
4-year Degree	19 (31.7%)	7 (24.1%)	12 (38.7%)
Graduate or Professional Degree	19 (31.7%)	7 (24.1%)	12 (38.7%)
Cognitive Capacity (Mean, SD)			
Telephone Interview for Cognitive Status	35.7 (2.53)	35.2 (2.95)	36.3 (1.97)
Tumor Type and Grade (*n*, %)			
Glioblastoma, Grade IV	7 (11.7%)	5 (17.2%)	2 (6.5%)
Astrocytoma, Grade I	1 (1.7%)	1 (3.4%)	0 (0.0%)
Astrocytoma, Grade II	4 (6.7%)	0 (0.0%)	4 (12.9%)
Astrocytoma, Grade III	2 (3.3%)	1 (3.4%)	1 (3.2%)
Astrocytoma, Grade IV	5 (8.3%)	2 (6.9%)	3 (9.7%)
Oligodendroglioma, Grade II	13 (21.7%)	7 (24.1%)	6 (19.4%)
Oligodendroglioma, Grade III	0 (0.0%)	0 (0.0%)	0 (0.0%)
Meningioma, Grade I	15 (25.0%)	9 (31.0%)	6 (19.4%)
Meningioma, Grade II	5 (8.3%)	1 (3.4%)	4 (12.9%)
Meningioma, Grade III	0 (0.0%)	0 (0.0%)	0 (0.0%)
Other	8 (13.3%)	3 (10.3%)	5 (16.1%)
Tumor Hemisphere (*n*, %)			
Left	22 (36.7%)	12 (41.4%)	10 (32.3%)
Right	22 (36.7%)	11 (37.9%)	11 (35.5%)
Bilateral	6 (10.0%)	1 (3.4%)	5 (16.1%)
Midline	6 (10.0%)	4 (13.8%)	2 (6.5%)
Unknown	4 (6.7%)	1 (3.4%)	3 (9.7%)
Tumor Lobe (*n*, %)			
Frontal	32 (53.3%)	15 (51.7%)	17 (54.8%)
Temporal	9 (15.0%)	5 (17.2%)	4 (12.9%)
Frontal-Temporal	3 (5.0%)	0 (0.0%)	3 (9.7%)
Parietal	2 (3.3%)	1 (3.4%)	1 (3.2%)
Occipital	1 (1.7%)	0 (0.0%)	1 (3.2%)
Parietal-Occipital	1 (1.7%)	0 (0.0%)	1 (3.2%)
Midbrain	0 (0.0%)	0 (0.0%)	0 (0.0%)
Midbrain-Temporal	0 (0.0%)	0 (0.0%)	0 (0.0%)
Brainstem	2 (3.3%)	2 (6.9%)	0 (0.0%)
Cerebellum	5 (8.3%)	2 (6.9%)	3 (9.7%)
Unknown	5 (8.3%)	4 (13.8%)	1 (3.2%)
Treatment History (*n*, %)			
Surgery	45 (75.0%)	22 (75.9%)	23 (74.2%)
Needle Biopsy Only	1 (1.7%)	1 (3.4%)	0 (0.0%)
Total Resection	21 (35.0%)	10 (34.5%)	11 (35.5%)
Partial Resection	16 (26.7%)	7 (24.1%)	9 (29.0%)
Resection, Extent Unknown	7 (11.7%)	4 (13.8%)	3 (9.7%)
Fractionated Radiation	27 (45.0%)	12 (41.4%)	15 (48.4%)
Immunotherapy	4 (6.7%)	2 (6.9%)	2 (6.5%)
Chemotherapy	25 (41.7%)	11 (37.9%)	14 (45.2%)
Tumor Treating Fields	10 (16.7%)	7 (24.1%)	3 (9.7%)
Active Treatment (*n*, %)			
Chemotherapy	5 (8.3%)	2 (6.9%)	3 (9.7%)
Tumor Treating Fields	6 (10.0%)	4 (13.8%)	2 (6.5%)
Current Medication Exposure (*n*, %)			
Corticosteroids	7 (11.7%)	4 (13.8%)	3 (9.7%)
Anticonvulsants	17 (28.3%)	11 (37.9%)	6 (19.4%)
Stimulants	6 (10.0%)	1 (3.4%)	5 (16.1%)
Sedatives (OTC or RX)	24 (40.0%)	14 (48.3%)	10 (32.3%)
Sleep Variables (*n*, %)			
Insomnia (Mean, SD)	16.27 (4.68)	15.34 (5.18)	17.13 (4.05)
None (0-7)	2 (3.3%)	1 (3.4%)	1 (3.2%)
Sub-Threshold (8-14)	19 (31.7%)	14 (48.3%)	5 (16.1%)
Moderate Clinical (15-21)	32 (53.3%)	12 (41.4%)	20 (64.5%)
Severe Clinical (22-28)	7 (11.7%)	2 (6.9%)	5 (16.1%)
Fatigue (Mean, SD)	5.09 (2.00)	4.55 (1.93)	5.59 (1.96)
Mild (1-3)	17 (28.3%)	10 (34.5%)	7 (22.6%)
Moderate (4-7)	38 (63.3%)	18 (62.1%)	20 (64.5%)
Severe (8-10)	5 (8.3%)	1 (3.4%)	4 (12.9%)
Sleep Quality (Mean, SD)	11.50 (3.12)	11.28 (2.88)	11.71 (3.36)
Good (0-5)	2 (3.3%)	1 (3.4%)	1 (3.2%)
Poor (6+)	58 (96.7%)	28 (96.6%)	30 (96.8%)

CBT-I = Cognitive Behavioral Therapy for Insomnia, TAU = Treatment as Usual, SD = Standard Deviation, OTC = Over the Counter, RX = Prescription.

### Feasibility of Longitudinal Data Collection

#### Patient-Reported Outcome Measures

At Week 0, 89% (62/70) of enrolled participants completed PROMs. Among randomized participants, 73% (44/60; CBT-I: 79% [23/29], TAU: 68% [21/31]) completed PROMs at Week 6 and 68% (41/60; CBT-I: 69% [20/29], TAU: 68% [21/31]) at Week 18.

#### Accelerometer

At Week 0, 100% (70/70) of enrolled participants agreed to accelerometer participation. Among randomized participants, 73% (44/60; CBT-I: 86% [25/29], TAU: 61% [19/31]) agreed at Week 6 and 60% (36/60; CBT-I: 55% [16/29], TAU: 64% [20/31]) at Week 18. USPS shipping issues occurred across all timepoints (Week 0: *n *= 5; Week 6: *n *= 1; Week 18: *n *= 10), preventing data collection. Among participants who agreed and successfully returned devices (baseline *n *= 65; post-intervention *n *= 43; follow-up *n *= 26), accelerometer adherence (≥3 days worn) was 87% (57/65) at Week 0, 93% (CBT-I: 92% [23/25]; TAU: 95% [18/19]) at Week 6, and 100% (CBT-I: 100% [12/12]; TAU: 100% [14/14]) at Week 18.

#### Neurocognitive Testing

At Week 0, 90% (63/70) of enrolled participants completed neurocognitive testing. Among randomized participants, 65% (39/60; CBT-I: 67% [19/29], TAU: 65% [20/31]) completed testing at Week 6, with 3 declining participation and 11 were LTFU. At Week 18, 53% (32/60; CBT-I: 55% [16/29], TAU: 52% [16/31]) completed testing, with 6 declining participation and 15 were LTFU.

### Acceptability of CBT-I

#### Intervention Retention and Session Attendance

Among participants randomized to CBT-I, 86% (25/29) completed all 6 sessions of the 6-week intervention. Overall, 11% of attended sessions were make-up visits (17/160). Fourteen participants required at least 1 make-up session, and 2 required 2 make-up sessions during the intervention period.

#### Intervention Satisfaction at Post-Intervention (Week 6)

All participants who completed CBT-I reported improved sleep (100%; 23/23; mean [SD], 7.26 [1.86]; range, 3-10). Most reported moderate-to-high success implementing CBT-I components (96%; mean score, 7.43; range, 3-10). Satisfaction with the telehealth format was high (91%), with few concerns regarding technology (96%), communication (91%), or privacy (91%). Most participants felt understood and reported that their questions were adequately addressed by the interventionist (95%). Participants also endorsed several benefits of telehealth, including reduced travel time (78%), reduced anxiety (39%), reduced infection risk (26%), and fewer distractions (39%). For future delivery, 70% recommended telehealth, 26% reported no preference, and 4% preferred in-person visits. Most participants indicated they would complete the program again (87%), and all would recommend it to others with brain tumors and insomnia (100%).

### Acceptability of Study Procedures at Follow-Up (Week 18)

Nearly all randomized participants reported that the longitudinal study procedures were acceptable (98%; 40/41). PROMs were considered acceptable with respect to ease (85%; *M *= 4.34, SD = 0.73, range 3-5), time commitment (78%; *M *= 4.05, SD = 0.97, range 1-5), and effort required (85%; *M *= 4.24, SD = 0.87, range 2-5). Neurocognitive testing was also largely rated as acceptable in terms of ease (73%; *M *= 3.93, SD = 0.91, range 1-5), time commitment (90%; *M *= 4.29, SD = 0.64, range 3-5), and effort required (78%; *M *= 4.02, SD = 0.85, range 1-5). Most participants found wearing the wrist-worn accelerometer acceptable (95%; *M *= 4.49, SD = 0.60, range 3-5) and reported minimal difficulty with returning the accelerometer using USPS (93%; *M *= 4.41, SD = 0.63, range 3-5). Regarding future accelerometer return procedures, 60% anticipated no difficulty using a nearby FedEx or UPS drop box, 35% anticipated some difficulty without affecting return capability, and 5% anticipated significant difficulty. Most participants agreed that the compensation ($25) at each data collection timepoint was adequate (83%).

### Meaningful Signal Detection

Intervention effects were calculated for participants who completed Week 0, 6, and 18 PROMs (Table 4; Figure 2). Of the 3052 total individual observations, 13 (0.43%) were missing. Imputation was not conducted for these exploratory analyses.

**Figure 2.**
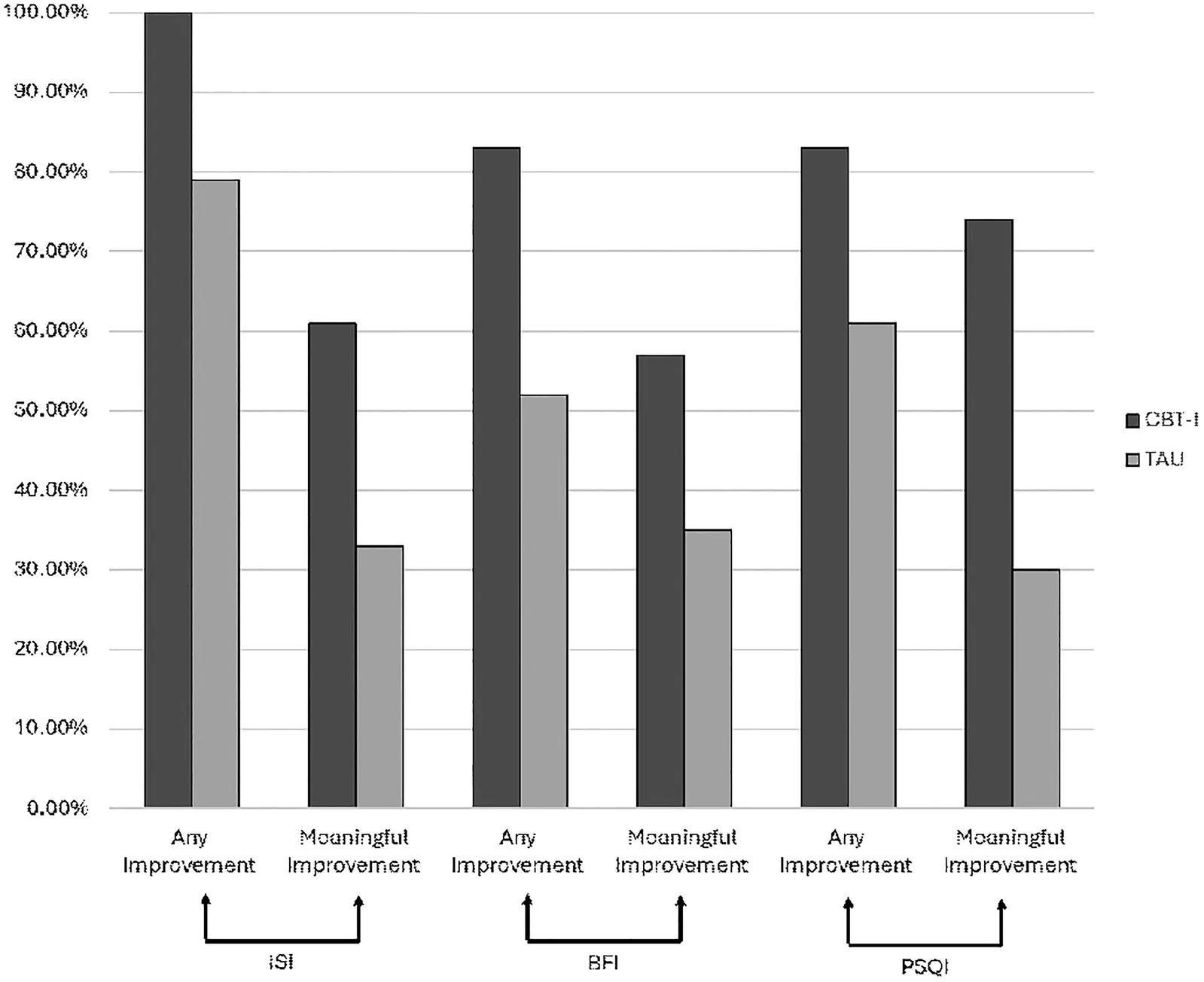
Percentage of participants achieving any and meaningful improvement in patient-reported insomnia severity (ISI), fatigue (BFI), and sleep quality (PSQI) at week 6.

**Table 4.** Insomnia, fatigue, and sleep quality scores by arm and timepoint

	Baseline Week 0 M (SD) *n* = 44	Post-Intervention Week 6 M (SD) *n* = 44	Δ Week 6-0 M (SD)	Cohen’s *d* (95% CI)	Follow-Up Week 18 M (SD) *n* = 41	Δ Week 18-6 M (SD)
Insomnia (ISI)						
CBT-I	15.13 (4.58)	6.91 (3.64)	−8.22 (5.04)	−1.63 (−2.25, −1.01)	7.55 (4.65)	0.75 (4.33)
TAU	16.96 (3.90)	12.92 (5.17)	−4.04 (5.45)	−0.73 (−1.18, −0.28)	12.52 (5.13)	−0.29 (2.94)
Fatigue (BFI)						
CBT-I	4.41 (1.65)	2.96 (2.08)	−1.45 (2.04)	−0.69 (−1.15, −0.24)	3.38 (2.68)	0.57 (1.99)
TAU	5.17 (1.83)	4.34 (2.11)	−0.83 (2.07)	−0.39 (−0.82, 0.03)	4.26 (2.52)	−0.23 (1.25)
Sleep Quality (PSQI)						
CBT-I	11.22 (2.80)	6.35 (3.05)	−4.87 (4.32)	−1.13 (−1.65, −0.60)	7.20 (3.40)	0.80 (2.73)
TAU	11.43 (3.49)	10.13 (4.03)	−1.30 (3.92)	−0.33 (−0.75, 0.09)	10.05 (3.84)	−0.48 (2.93)

M = Mean, SD = Standard Deviation, Δ = change, CBT-I = Cognitive Behavioral Therapy for Insomnia, TAU = Treatment as Usual, ISI = Insomnia Severity Index, BFI = Brief Fatigue Inventory, PSQI = Pittsburgh Sleep Quality Index.

#### Insomnia

Among the 23 CBT-I participants who completed the insomnia PROM at Weeks 0 and 6, all (100%) demonstrated decreased ISI scores, with 61% (14/23) meeting criteria for a MID (ΔISI ≥6; Cohen d= −1.63). Among participants retained at Week 18, 70% demonstrated sustained or additional reductions in ISI scores. In the TAU group, 79% (19/24) of participants who completed the insomnia PROM at Weeks 0 and 6 demonstrated decreased ISI scores, with 33% (8/24) meeting MID criteria (Cohen d= −0.73). Among those retained at Week 18, 52% demonstrated sustained or additional reductions in ISI scores.

#### Fatigue

Among the 23 CBT-I participants who completed the fatigue PROM at Weeks 0 and 6, 83% (19/23) demonstrated decreased BFI scores, with 57% (13/23) meeting criteria for a MID (ΔBFI ≥1.33; Cohen d= −0.69). Among those retained at Week 18, 26% demonstrated sustained or additional reductions in BFI scores. In the TAU group, 52% (12/23) of participants who completed the fatigue PROM at Weeks 0 and 6 demonstrated decreased BFI scores, with 35% (8/23) meeting MID criteria (Cohen d= −0.39). Among those retained at Week 18, 25% demonstrated sustained or additional reductions in BFI scores.

#### Sleep Quality

Among the 23 CBT-I participants who completed the sleep quality PROM at Weeks 0 and 6, 83% (19/23) demonstrated decreased PSQI scores, with 74% (17/23) meeting criteria for a MID (ΔPSQI ≥3; Cohen d= −1.13). Among participants retained at Week 18, 35% demonstrated sustained or additional reductions in PSQI scores. In the TAU group, 61% (14/23) of participants who completed the sleep quality PROM at Weeks 0 and 6 demonstrated decreased PSQI scores, with 30% (7/23) meeting MID criteria (Cohen d= −0.33). Among those retained at Week 18, 52% demonstrated sustained or additional reductions in PSQI scores.

## Discussion

The primary aims of this Phase IIc randomized controlled study were to evaluate the feasibility and acceptability of CBT-I and study procedures in adults with PBT and comorbid insomnia. Additionally, we explored meaningful intervention effects on patient-reported insomnia severity, fatigue, and sleep quality compared with TAU. Overall, our findings provide strong support for the feasibility and acceptability of CBT-I in this population, highlight longitudinal data collection as an area for refinement ahead of a future large-scale efficacy trial, and reveal a clear and meaningful signal that CBT-I may lead to improvements in insomnia severity among patients with PBT.

### Feasibility of Recruitment

Overall findings provide strong support for recruitment feasibility, as pre-specified eligibility and enrollment benchmarks were exceeded. However, among individuals who expressed interest, 53% were successfully contacted for screening, which was below our target metric. This may be due in part to the cohort-based nature of the current trial: it is possible that individuals reached out when they perceived the greatest need or when they first heard about the study but lost interest before the study team was ready to launch a new cohort. In addition, the ability to successfully connect with interested individuals with brain tumors may be influenced by the fluctuating symptom burden associated with neuro-oncologic conditions. It is important to note that once direct contact was established, eligibility (96%) and consent (86%) rates were high, underscoring strong interest in behavioral sleep interventions within this population. Given that the most successful recruitment outlets for yielding consented participants were social media and patient-facing webinars, direct engagement with this patient population appears to be an important consideration when developing a recruitment plan. At the same time, referrals from local and national provider networks yielded more than a quarter of our enrolled sample and appear to remain a critical component of a comprehensive recruitment plan.

### Feasibility Longitudinal Data Collection

The feasibility of longitudinal study procedures varied by the type of assessment. Across all timepoints, PROM data collection was feasible for the overall sample, meeting or exceeding all established benchmarks. These findings were also largely consistent across study arms. Although the TAU arm narrowly missed the 70% completion benchmark at the post-intervention timepoint, this shortfall reflected the absence of a single participant and does not meaningfully detract from the overall feasibility of PROM data collection procedures.

Agreement to accelerometer participation met feasibility benchmarks across all timepoints. However, unanticipated USPS shipping challenges resulted in both device and data loss. After accounting for declined participation and mailing challenges, accelerometer adherence among participants who successfully received devices exceeded established benchmarks across all three timepoints. Prior studies in PBT populations^54^^,^^55^ have also reported high adherence rates (72%-80%) for wrist-worn wearable devices over 1- and 4-month monitoring periods; however, these studies used different data collection procedures, such as continuous device replacement at 14-day intervals or ongoing device monitoring without replacement, but requiring continual coordinator oversight. Although alternative designs—such as continuous monitoring—might have improved feasibility metrics if baseline participation had been maintained, they do not necessarily guarantee higher adherence. Notably, our findings indicate that when participants agreed to this procedure and successfully received the device, adherence was exceptionally high, yielding usable accelerometer data. These results suggest that continuous wearable monitoring, rather than discrete data collection periods requiring repeated device shipment, may represent a more feasible design for future trials. Integrating a wrist-worn device capable of synchronizing with a centralized data management platform could also allow study coordinators to monitor device malfunctions, battery depletion, and nonadherence in real time, thereby minimizing data loss. However, such approaches may increase participant burden, require more intensive monitoring and communication regarding device charging and compliance, provide limited extractable data, and incur substantially increased logistical costs.^56-58^ Despite these challenges, similar strategies have been shown to improve wearable adherence in similar populations.^54^

Longitudinal neurocognitive testing also presented challenges. Baseline neurocognitive data collection exceeded feasibility expectations, and post-intervention completion rates (65%) approached the established 70% benchmark. However, follow-up neurocognitive testing (Week 18) again fell short of feasibility targets, with only 53% completion relative to the predefined 60% benchmark. While attrition in longitudinal neurocognitive assessment is common in neuro-oncology research, the rates observed at our 18-week timepoint exceed those reported in recent feasibility studies.^59^^,^^60^ Explicit reasons for missing neurocognitive data (eg participant-reported reasons for declining participation or lack of response to email communication) were not systematically captured in this study, limiting our ability to identify factors contributing to reduced feasibility at this timepoint. Patient fatigue has historically been cited as a primary reason for missed neurocognitive testing in brain tumor populations;^61^^,^^62^ however, cognitive-behavioral therapy has been shown to improve fatigue in individuals with brain tumors,^63^ which would hopefully have guarded against some of this specific barrier to participation in our study, specifically to those in the CBT-I randomized group. Other documented contributors to neurocognitive testing attrition include unexplained missed clinic appointments, illness-related factors, and refusal of testing procedures.^63^ Further, in the current study design, participants received compensation at each timepoint regardless of how many data components were completed (eg $25 for completion of 1, 2, or all 3 components). Although most participants reported that compensation was adequate, restructuring compensation to be distributed by each individual data component (eg PROMs, accelerometry, and neurocognitive testing) for a total of up to $75 per timepoint may incentivize continued engagement and improve adherence across longitudinal assessments.

### Acceptability of CBT-I and Study Procedures

Participant satisfaction with the intervention was exceptionally high. Consistent with—and exceeding—findings from our Phase II single-arm trial,^32^ all participants (100%) in the current trial believed their sleep improved following CBT-I and would unanimously recommend the intervention to peers with PBT experiencing insomnia. Participants also reported successful implementation of CBT-I strategies into their daily lives. Similarly, satisfaction with the telehealth delivery model was robust. Participants reported minimal barriers related to technology, communication, or privacy. The majority explicitly recommended that telehealth delivery is maintained in future trials, supporting its acceptability, feasibility, and scalability. Despite the high intervention retention rate (86%), nearly half of participants in the 6-week group telehealth CBT-I intervention required at least 1 make-up session. Although this was feasible within the schedules of our study interventionists, it may be more difficult in a larger-scale trial or clinical practice, and it highlights the necessity of flexibility when delivering behavioral interventions to medically ill populations. Future trials should therefore intentionally incorporate a modest buffer period to accommodate health- or treatment-related disruptions, as well as planned travel or vacation, to maximize engagement and completion without compromising intervention fidelity. Overall, study procedures were highly acceptable to participants, with nearly all reporting that PROMs, accelerometer use, and neurocognitive testing were manageable.

### Meaningful Signal Detection

Consistent with prior findings across oncology populations^63^ and our earlier single-arm trial in patients with PBT,^32^ the CBT-I intervention demonstrated meaningful improvements in insomnia severity, fatigue, and sleep quality. This Phase IIc pilot trial extends science by providing a randomized comparison of CBT-I vs. TAU in neuro-oncology and demonstrating between-group differences across all outcomes. From Week 0 to Week 6, CBT-I produced a strong therapeutic signal, and the proportion of participants achieving meaningful improvements was consistently greater in the CBT-I group—at times nearly doubling that demonstrated by TAU. These gains were supported by medium-to-large within-group effect sizes for CBT-I (*d *=* −*0.69 to *−*1.63), exceeding the small-to-­moderate effects in TAU (*d *=* −*0.33 to *−*0.73). Sustained benefit further supported insomnia severity as the most robust treatment outcome, as 70% of CBT-I participants maintained or achieved continued reductions in insomnia severity compared to 52% in TAU at Week 18. Although sustained improvement patterns were comparable across groups for fatigue severity and favored TAU for sleep quality, the magnitude and consistency of the reductions in insomnia severity highlight this outcome as the most robust treatment-response endpoint of CBT-I in this population. Given the high prevalence of insomnia in patients with brain tumors and its broad downstream effects, including increased emotional distress and impaired quality of life,^1-8^ insomnia severity represents a clinically meaningful and high-yield primary endpoint.

Insomnia in patients with PBT is likely multifactorial, arising from the combined effects of psychological distress, treatment-related factors, medication use (eg corticosteroids and antiseizure medications), neurologic symptoms, and disruption of neural networks involved in sleep regulation. While CBT-I is designed to address the behavioral and cognitive processes that perpetuate chronic insomnia regardless of the initiating cause, future research should investigate whether clinical and neuroanatomical factors, including tumor location and network involvement, moderate treatment response. The larger sample afforded by our future Phase III efficacy trial will enable prospective evaluation of these potential moderators.

### Limitations

The results of the current study must be interpreted in the context of several limitations. First, this study was not adequately powered to reliably detect statistically significant between-group differences. While our sample was relatively heterogenous with respect to race/ethnicity, this is representative of the broader PBT population. Most of our samples were highly educated, which may limit generalizability. Although participant-reported adherence to CBT-I guidelines was collected, interventionist-reported adherence was not, which may have differed substantially. Lastly, we were unable to collect acceptability data from participants who did not complete follow-up study procedures, which may introduce bias, as it is possible that these participants chose not to complete activities precisely because they found them to be unacceptable. However, these limitations can be addressed and/or further examined in a larger-scale efficacy trial with targeted recruitment of those who may be most at risk of drop-out, including historically underserved and under-resourced populations.

## Conclusion

Findings from this NIH ORBIT Phase IIc randomized controlled study provide strong support for progression to a Phase III efficacy trial of CBT-I for patients with PBT and comorbid insomnia. Recruitment feasibility was robust, demonstrated by high eligibility and enrollment rates. Feasibility of longitudinal data collection was variable; while PROMs data met expectations across timepoints, refinements to both the accelerometer and neurocognitive testing procedures will be necessary if these outcomes are retained in a fully powered efficacy trial. The CBT-I intervention was highly acceptable, with patients consistently reporting improvements in sleep and strongly endorsing the program, underscoring its clinical relevance in this population. The meaningful signal detections demonstrate a clear and clinically relevant signal for CBT-I, providing justification for advancement to a fully powered Phase III efficacy trial with insomnia severity at Week 6 as the primary endpoint.

## Data Availability

The data that support the findings of this study are available from the corresponding author, ARL, upon reasonable request.
